# Diagnosis of Allergy to Mammals and Fish: Cross-Reactive vs. Specific Markers

**DOI:** 10.1007/s11882-017-0732-z

**Published:** 2017-08-22

**Authors:** Christiane Hilger, Marianne van Hage, Annette Kuehn

**Affiliations:** 1 Department of Infection and Immunity, Luxembourg Institute of Health, 29, rue Henri Koch, L-4354 Esch-sur-Alzette, Luxembourg; 2 Immunology and Allergy Unit, Department of Medicine Solna, Karolinska Institutet, and Karolinska University Hospital, Stockholm, Sweden

**Keywords:** Allergy diagnosis, Allergen component, Cross-reactive allergen, Cross-sensitization, Fish allergy, Furry animal allergy

## Abstract

**Purpose of Review:**

Allergen extracts are still widely used in allergy diagnosis as they are regarded as sensitive screening tools despite the fact that they may lack some minor allergens. Another drawback of extracts is their low specificity, which is due to the presence of cross-reactive allergens. Progress in allergen identification has disclosed a number of allergenic molecules of homologous sequence and structure which are present in different animal species. This review summarizes recent advances in mammalian and fish allergen identification and focuses on their clinical relevance.

**Recent Findings:**

Serum albumins and parvalbumins are well-known animal panallergens. More recently several members of the lipocalin family were found to be cross-reactive between furry animals whereas in fish, additional allergens, enolase, aldolase and collagen, were found to be important and cross-reactive allergens. New epidemiological studies have analysed the prevalence and clinical relevance of mammalian and fish components.

**Summary:**

Primary sensitization can be distinguished from cross-sensitization by using marker allergens. Although substantial progress has been made in allergen identification, only few markers are commercially available for routine clinical practice.

## Introduction

Allergy diagnosis is still mainly based on allergen extracts. Skin prick test solutions as well as the majority of assays used for in vitro diagnosis are composed of extracts. They are relatively easy to produce and although they are difficult to standardize, they are essential diagnostic tools. However, they also have some serious drawbacks such as variable allergen content, underrepresentation of minor allergens, potential contamination by other allergen sources and very importantly, cross-reactivity among allergens present in different extracts which precludes a differential diagnosis [1]. Single allergen molecules or components have found their way into IgE-based diagnostics, but their proven utility in allergy diagnosis still needs to be implemented in daily clinical practice.

The last two decades have brought enormous progress in allergen identification and characterization. About 870 allergens are registered in the World Health Organization and International Union of Immunological Societies (WHO/IUIS) Allergen Nomenclature Sub-Committee database (http://www.allergen.org) based on evidence of allergenicity and many more have been described in scientific publications. We are now aware of the fact that homologous proteins are present in different allergen sources. The pathogenesis-related (PR) protein family 10, the non-specific lipid transfer proteins (nsLTP) and profilins are well-known panallergens in pollen and plant foods [2••]. Tropomyosin is the hallmark of IgE cross-reactivity among invertebrates such as shellfish, molluscs and arthropods [2••]. In vertebrates, the only known panallergens are parvalbumins, the major fish allergens, and serum albumins, minor allergens of mammals [2••]. More recently, additional cross-reactive allergens have been found in fish and furry animals [3•, 4, 5, 6•, 7, 8]. The relevance of these cross-reactive allergens will be reviewed in this chapter.

**Fig. 1:**
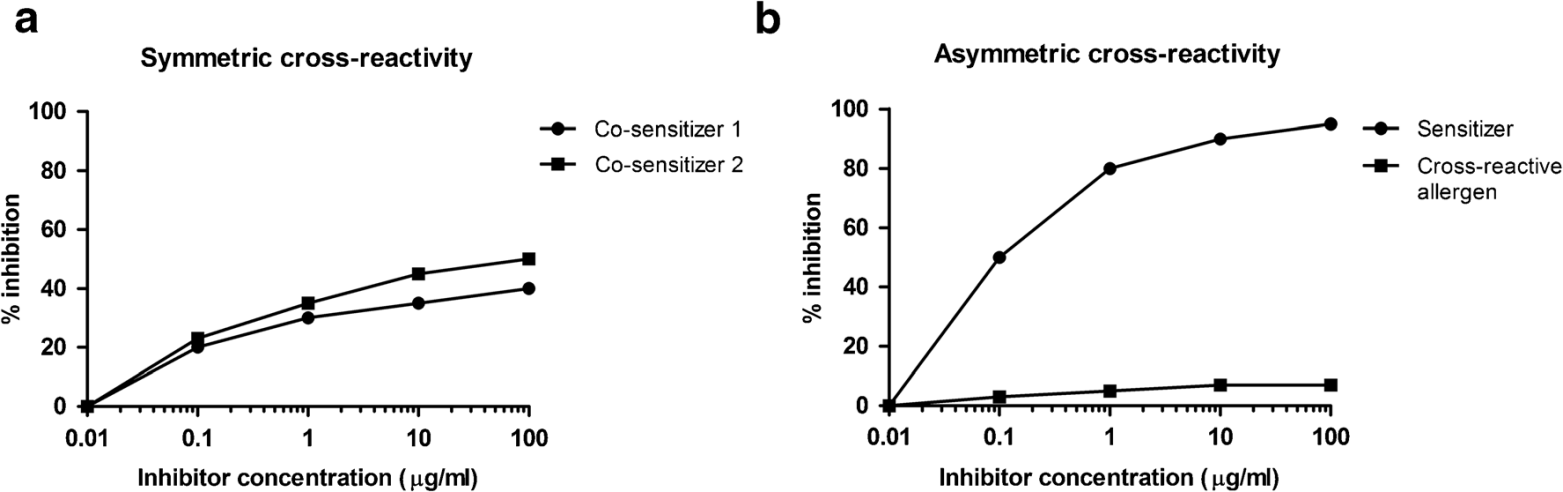
Symmetric versus asymmetric cross-reactivity. **a** Allergen 1 and 2 are co-sensitizers. Inhibition of IgE binding to allergen 1 can be partially inhibited by allergen 2 (*squares*); reciprocally allergen 1 also partially inhibits IgE binding to allergen 2 (*circles*).**b** Allergen 1 is the primary sensitization source (*sensitizer*). The sensitizer (*circles*) completely inhibits binding to allergen 2 (cross-reactive allergen) whereas allergen 2 (s*quares*) is not able to inhibit IgE binding to allergen 1

The prediction of allergen cross-reactivity is commonly achieved by protein sequence comparisons. As a general rule, allergens with less than 50% amino acid identity are rarely cross-reactive [9]. Since IgE antibodies recognize protein structures, another approach could be to predict conformational epitopes on allergens, to compare their protein surfaces and to identify exposed potential common epitopes [10]. Unfortunately, the number of resolved allergen structures is still limited. In addition, food allergens can be modified and degraded upon food processing and digestion. AllergenOnline provides tools for the identification of proteins that may present a potential risk of allergenic cross-reactivity (http://www.allergenonline.org/). Three criteria are used, (i) full-length alignment with known allergens where a sequence identity of > 50% will indicate a potential cross-reactivity, (ii) use of a sliding window of 80 amino acid segments to find identities of > 35% and (iii) search for an exact match of 8 amino acids [11•]. However, the predictive value of the short exact match in the absence of longer identity alignments is questionable and predicted cross-reactivity needs to be confirmed by IgE-inhibition assays. Cross-reactivity can be symmetric or asymmetric [12]. In the first case, both allergens have sensitized the patient and some IgE antibodies are directed to specific epitopes, others to common epitopes. Each allergen is inhibiting binding of IgE to the other allergen to some extent (Fig. 1a). In the second case, one allergen (allergen 1) is the primary sensitization source and the patient has not been in contact with the second allergen (allergen 2) or did not produce specific IgE antibodies against this allergen. In that situation, allergen 1 can block binding of IgE to allergen 2, but allergen 2 is not able to inhibit IgE binding to allergen 1 and it can be deduced that allergen 1 is the primary sensitizing molecule (Fig. 1b).

Prediction of cross-reactivity is difficult as IgE antibody responses are polyclonal. Some IgE will be directed against unique epitopes, others will bind to cross-reactive epitopes. The fraction of these cross-reactive IgE is patient-dependent. The size of this fraction and the affinity of the cross-reacting antibodies probably play a role in the generation of clinical symptoms [12]. Not all IgE cross-reactions however translate into clinical cross-reactivity.

## Allergy to Mammals in Domestic and Occupational Environments

Mammalian animals are an important source of indoor allergens and they are considered as risk factors for the development of allergic rhinitis [13•]. They are very popular as pets and are present in up to 35% of European and up to 60% of US households. A German study analysed sensitization rates to inhalant and food allergens in more than 7000 healthy adults. A sensitization rate of 10% was recorded to animal epithelia, with a higher sensitization of 15.4% observed in the group of young adults (18–29 years) [14]. Specific IgE to cat and dog were 7.0% and 3.5% to horse. A survey of more than 12,000 German children found a sensitization rate of 12.6% to animals. The prevalence increased from 5.7% in the 3–6 years group to 11.5% in the group of 7–10 years and reached 17.2% in the 14-17 years group [15]. The Global Asthma and Allergy European Network (GAL^2^EN) has studied patients that visited an allergy centre for a suspected allergic reaction to inhalant allergens [16]. Sensitization rates were found to vary greatly over the 14 participating countries, with highest sensitization levels in Denmark (56% for dog, 49.3% for cat) and lowest in Austria, Belgium and Italy (< 20%). Mean prevalence for Europe was 27.2% for dog and 26.3% for cat. The Swedish BAMSE study, an unselected population-based birth cohort study of more than 4000 children, reported more than a triplication of sensitization rates to cat, dog and horse from 4 to 16 years, reaching 19.0% for cat, 22.6% for dog and 10.6% for horse [2••, 17].

Sensitization to animal allergens also plays a role in occupational settings. A recent Iranian study on 100 veterinarians and laboratory animal workers showed positive skin prick test to animal dander in 36% of the individuals working with animals versus 10% of the controls [18]. Forty-four percent of the veterinary students and 57% of the veterinarians showed work-related symptoms such as asthma, rhinitis, conjunctivitis and cutaneous symptoms. In laboratory animal workers, allergy to rodents is an important occupational disease and can affect between 11 and 44% of the exposed personnel [19]. Numerous studies have analysed exposure to rodent allergens in animal facilities [20•]. As exposure is dependent on the room, the job and the individual task, specific measures to reduce or to avoid allergen exposure are recommended. The use of individually ventilated cages has reduced ambient allergen levels up to 250-fold compared to the use of open cages [21•].

One drawback of all these epidemiological studies is that they are based either on skin prick tests or determination of specific IgE to animal dander. As these extracts contain a number of cross-reactive molecules, they do not allow to precisely determine sensitization to a specific animal, which may lead to an overestimation of sensitization rates to a given animal.

The availability of cat and dog allergen components has driven new epidemiological studies in order to analyse the prevalence and clinical relevance of specific IgE directed against individual allergens. A Swedish population-based cohort study of 696 children found a sensitization rate of 37.2% to furry animals. Asthma symptoms upon animal contact were significantly associated with specific IgE to cat allergens Fel d 1 and Fel d 4 for cat-allergic children, and with co-sensitization to dog allergens Can f 5 and Can f 1/2 in dog-allergic [22]. Asthma was also associated with higher IgE levels and the number of positive components from the same animal. In a random sample of 779 children from the Swedish BAMSE study, the longitudinal analysis from 4 to 16 years showed an increase of sensitization to individual cat or dog allergens but also an increase of co-sensitization to several components of the same animal and of co-sensitization to cat and dog allergens [23•]. Polysensitization at 4 years was a predictive risk marker for the development of allergic symptoms at 16 years. The determination of patient-specific IgE profiles will allow to establish predictive risk markers and to develop strategies for therapeutic intervention.

## Mammalian Allergens

### Major Allergen Families

Mammalian allergens belong to few protein families (Table 1) [24•]. The lipocalin protein family represents the largest group of allergens [25•]. The majority of known animal allergens are lipocalins and a number of them are classified as major allergens. They are small extracellular proteins characterized by a common tertiary structure composed of a central β-barrel composed of eight anti-parallel β-strands. Mammalian lipocalin allergens are odorant and pheromone binding proteins that carry small hydrophobic molecules in their internal binding pocket formed by the barrel. They are present in urine, saliva and animal dander. As they easily stick to particles, they become airborne and are transported to public places such as schools and day care centres [20•, 26].

**Table 1:**
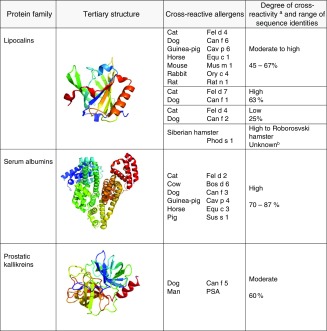

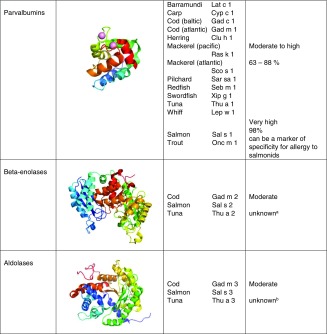
Cross-reactive molecules in mammals and fish

Tertiary structures are represented as ribbon models. N-terminal end in blue, C-terminal in red. Lipocalins are represented by Equ c 1 (1EW3), serum albumins by Equ c 3 (4F5U), prostatic kallikreins by human prostate-specific antigen (2ZCH), parvalbumins by Gad m 1 (2MBX), beta-enolase by human beta-enolase (2XSX) and aldolases by fructose 1,6-bisphosphate aldolase from rabbit muscle (3B8D)

^a^For most of the molecules, cross-reactivity has been determined experimentally, for some combinations, classification is based on expected cross-reactivity

^b^Sequences of allergenic molecules are not completely known, therefore, no sequence identity can be deduced

Serum albumins are the second most frequent animal allergens. They are large globular proteins and a major component of animal and human plasma. They are also present in animal dander and fluid such as urine, saliva and milk. They are structurally highly conserved and share also high sequence identities of 70–87% [27]. Although highly cross-reactive, they are considered as minor allergens.

Fel d 1 and Ory c 3 belong to the secretoglobin family. Both allergens share a conserved tertiary structure, a glycosylated heterodimer composed of two small proteins. Sequence identity between both allergens is very low and so far, no IgE cross-reactivity has been observed [28]. Both secretoglobins are synthesized in the salivary glands, Fel d 1 is also expressed in the skin.

Two allergens belong to the latherin family, Equ c 4 and Fel d 8 [29, 30]. Equ c 4 was shown to have surfactant properties and it is supposed to play a role in evaporative cooling by horse sweat. Although Equ c 1 is not a latherin, but a lipocalin, it has also been found to lower surface tension of water [31]. Fel d 3, a minor allergen, belongs to the cystatin family that are cysteine protease inhibitors [32]. Can f 5 is a major dog allergen. It is a prostatic kallikrein found in male dog urine [33].

### Cross-Reactive Mammalian Allergen Components

#### Serum Albumins

Serum albumins are cross-reactive allergens of animal dander, meat and milk. Seven components are registered in the WHO/IUIS Allergen Nomenclature Sub-Committee database: Bos d 6 (bovine), Can f 3 (dog), Cav p 4 (guinea-pig), Equ c 3 (horse), Fel d 2 (cat) and Sus s 1 (pig). Gal d 5, chicken serum albumin, displays a rather low sequence identity to the mammalian serum albumins (42–46%). Cross-reactivity is estimated to be rare, but has been confirmed in a case report [34]. In addition to the officially named serum albumins, a number of albumins from different animals have been shown to bind IgE and to be cross-reactive [35].

In the context of respiratory allergens, serum albumins are considered as minor allergens without clinical relevance. In different study populations, IgE prevalence to cat or dog serum albumin has been estimated from 14 to 50%. A monosensitization to Fel d 2 or Can f 3 seems to be very rare. The occurrence of specific IgE to Fel d 2 without sensitization to Fel d 1 could be a marker of cross-reactivity to another animal and the primary sensitization source should be searched for. As sensitization to serum albumins is always seen in combination with specific IgE directed against major allergens, the clinical relevance of serum albumins is still unclear. A few cases of clinical reactions to Bos d 6 have been reported. Laboratory workers are often exposed to Bos d 6 as it is widely used in biochemical and immunological assays. Two cases of occupational asthma have been attributed to Bos d 6 inhalation in laboratory workers [36, 37]. The use of Bos d 6 in cell culture media has provoked severe anaphylactic reactions upon artificial insemination [38]. High titers of IgE to Fel d 2 have been associated with atopic dermatitis in cat-allergic children [39].

The role of serum albumins seems to be more important as food allergens. They are major components of milk and meat. Serum albumin constitutes about 1% of the total protein content of bovine milk (0.1–0.4 g/l) [40]. Bos d 6 is thermolabile and it has been shown that boiling milk for 10 min abolished skin prick test responses in patients sensitized to Bos d 6. Together with β-lactoglobulin (Bos d 5), α-lactalbumin (Bos d 4) and the caseins (Bos d 8), it is a major milk allergen. In a cohort of 80 children with immediate reactions to milk confirmed by double-blind, placebo-controlled food challenge (DBPCFC), 61.3% had specific IgE to Bos d 6 [40]. In children with clinical reactivity to beef, Bos d 6 has been proposed as a marker of cow’s milk allergy. In a study on 28 children with DBPCFC allergy to beef, 92.9% had also a clinical reactivity to cow’s milk [41].

Clinical cross-reactivity between serum albumins from animal dander and meat can occur between different species, but the association between cat and pork is the most frequent one [42]. Pork-cat syndrome was first described in France in the 1990s [43], later it has been reported in Luxembourg and in the USA [44, 45]. In a group of 39 highly sensitized cat-allergic patients, we have found that 23% had specific IgE to Fel d 2, cat serum albumin and more than half of them had specific IgE to Sus s 1, porcine serum albumin [44]. One out of three patients experienced clinical symptoms upon ingestion of pork meat. Overall, it can be estimated that up to 3% of patients highly sensitized to cat may develop symptoms upon ingestion of pork ham or sausages, which are raw meat. Most patients tolerate well cooked meat or milk as serum albumins are thermolabile.

#### Lipocalins

Animal lipocalins are structurally well conserved molecules, but they have very divergent sequences with amino acid sequence identities often as low as 20–30%. They were therefore considered as specific markers for diagnosis of the sensitization source. Recent progress in allergen cloning and characterization has disclosed new family members that display much higher sequence identities among lipocalins [4, 25]. The group of major lipocalin allergens, Equ c 1, Fel d 4, Can f 6, Cav p 6, Ory c 4, Rat n 1 and Mus m 1 show identities between 45 and 67% [2••]. IgE cross-reactivity has been well documented by ELISA inhibition experiments between Fel d 4 and Can f 6 as well as between Can f 6, Fel d 4 and Equ c 1 [5, 6•]. A weak cross-reactivity between Equ c 1 and Mus m 1 has also been confirmed by inhibition studies [7]. Cross-reactivity has also been observed between rat and mouse urine using RAST inhibition and immunoblotting experiments [46]. As Mus m 1 and Rat n 1 are the main components of rodent urine, it is likely that they were the cross-reacting molecules. They share 64% of sequence identity. While the lipocalins described above constitute a large group of allergens with high sequence identities, there also exist some isolated pairs of lipocalins which share substantial identity. This is the case for Fel d 7 and Can f 1. In Swedish cat-sensitized patients, 46% had specific IgE to Fel d 7 and 92% of these had also specific IgE to Can f 1. Inhibition experiments showed a patient dependent IgE cross-reactivity between both lipocalins that share an identity of 63% [8]. Can f 1 was also found to cross-react with human tear lipocalin Lcn-1 [7]. A recently isolated allergen from Siberian hamster, Phod s 1 showed no cross-reactivity to European and golden hamster; however, IgE reactivity to Phod s 1 could be partially inhibited by salivary gland extracts of the Roborovski hamster [47]. Siberian and Roborovski hamster belong both to the Phodopus species and are more closely related to each other than to the golden and European hamsters. The allergen sequence of the Roborovski hamster is only partially known, but it seems to be highly identical to Phod s 1 [48].

Although sequence alignments of allergens allow to predict IgE cross-reactivity to some extent, they are not able to exclude cross-reactivity. Even small stretches of conserved residues, either in linear or conformational epitopes, may lead to IgE binding in some patients. Indeed, weak cross-reactivity has been shown between Can f 2 and Fel d 4 although they have a sequence identity of only 25%. Cross-reactivity was patient dependent, arguing for a limited number of cross-reactive epitopes [49]. Can f 4 has been reported to cross-react with a 23 kDa lipocalin present in bovine dander (sequence identity 35%) [50].

A number of lipocalins do not have high sequence identity to other known members of the family and so far, no relevant cross-reactivity has been reported. These are Cav p 2, Cav p 3, Mes a 1 and Bos d 2 [2••]. Others are not yet well characterized, such as Cav p 1, Equ c 2 and Ory c 1 for which only N-terminal sequences are available.

#### Other Cross-Reactive Allergens

Inhibition with Fel d 1 has been shown to reduce IgE binding to dog extracts in some patients that were allergic to cats and who also had specific IgE to dog dander [51]. Up to now, no Fel d 1-like allergen has been found in dog. A Fel d 1-like allergen has been isolated from rabbit hair and saliva, but no cross-reactivity to Fel d 1 could be detected [28].

Can f 5, a major dog allergen and a prostatic kallikrein, cross-reacts with human prostate-specific antigen and mediates vaginal reactions to human seminal plasma [33].

### Marker Allergens of Furry Animals

Fel d 1 is regarded as a good marker allergen for a primary sensitization to cat as it is specific for cat and has a high IgE prevalence. Although Fel d 1 is generally recognized by > 90% of cat-allergic patients [13•], there are also reports on patient cohorts in which specific IgE to Fel d 1 have a much lower prevalence [52, 53]. However, sensitization to cat components had a stronger prognostic value for new-onset of rhinitis and asthma in comparison to the determination of sensitization to cat extract [53]. For dog, Can f 1, Can f 2 and Can f 5 may be regarded as specific markers for sensitization to dogs. As neither of them reaches a sensitization rate above 90%, it is preferable to quantify specific IgE against all 3 components, taking into account that Can f 1 also displays some cross-reactivity with Fel d 7. If only Can f 1 is positive, sensitization to cat may be excluded by a negative test to Fel d 1.

The only available components for horse are Equ c 1 and Equ c 3. Although Equ c 1 was regarded as marker allergen, this must now be reconsidered as a high cross-reactivity with the cat and dog allergens Fel d 4 and Can f 6 has been demonstrated [6•]. The same applies for Mus m 1 and Rat n 1 as they are mutually cross-reactive and their high sequence identity to Ory c 4 (51–54%) and Fel d 4 (55% for Rat n 1, 49% for Mus m 1) could point to other potential cross-reactivities. For small furry animals, different allergens have been isolated and some of them, Cav p 2, Cav p 3, Ory c 3, Phod s 1 and Mes m 1, could be used as specific marker allergens for sensitization to guinea-pig, rabbit, dwarf and golden hamster respectively, but unfortunately they are not commercially available.

## Allergy to Fish, a Food and Respiratory Allergen

Fish is an important food which is commonly consumed all over the world, in westernized countries as of trends towards healthy and lean nutrition and in developing, coastal countries because of availability of locally produced fish products [54]. Fish consumption is steadily on the rise since the past decades. In 2016, the global per-capita consumption per year reached 20 kg for the first time, according to the Food and Agriculture Organization (FAO) of the United Nations (www.fao.org). The animal kingdom of fish is highly diverse, thousands of species are known and about 800 fishes are available on national and international food markets. Different fish species are eaten in many countries over the world. Tuna, salmon, cod, Alaska Pollock and herring are the top five food fishes in Europe. Salmon, tuna, Alaska pollock and cod are also commonly consumed in the USA, in addition to catfish, pangasius and tilapia. In the southern hemisphere, barramundi, sharks, flathead and Asian carps have also relevant market shares and thus, are of economic value [55].

Fish has a high benefit of nutritive value but unfortunately, fish is also counted among the most allergenic foods in the world. Fish allergy often develops during early childhood, but in contrast to milk or egg allergy, it usually persists beyond school age. Even genesis of this food allergy during adulthood is not exceptional. Fish allergy is a multi-organ disease with symptoms affecting the skin, the gastrointestinal and/or the respiratory tract and in a systemic way, even leading to an anaphylactic shock requiring emergency treatment and medication [55–57]. A number of epidemiological studies have so far focused on the prevalence of fish allergy, with variable outcome according the respective study design. Variable data has been shown based on self-report, skin-tests or food challenges. Previous meta-analysis estimated 0.6% of fish prevalence using self-report, 0.6% using skin test, 0.7% using IgE reactivity to fish extracts and 0.1% using diagnostic food challenges or clear medical history of fish allergy [58]. A recent, systematic review on prevalence-based literature inside and outside Europe revealed a much broader prevalence spectrum, ranging from 0 to 7% and pointing to country-specific as well as cohort-specific factors which is detailed below [59•].

Using questionnaires, the prevalence in Europe was found to vary in adults from 1.5% in Greece (20–54 years of age) to 0.2% in Denmark (22 years) [60]. In children, percentages reached from 7.0% in Finland (1 year), over 2.8% in the Eastern Mediterranean (6–9 years) to 0.0% in Israel (0–2 years) [61, 62]. In the USA, the prevalence ranged from 2.7% in adults to 0.2% in children [63, 64]. In Southeast Asia, fish allergy rates were reported for Thailand at 1.1% (3–7 years), for the Philippines at 4.3% (14–16 years) and for Hong Kong at 0.2% (2–7 years) [65–67].

Using diagnostic skin testing, allergic sensitizations to fish were reported in Europe on a scale from 2.9% in adults (25–74 year of age; Germany) to 2.7% in children (15–17 year of age; Finland). Combining medical history and sensitization analysis, the highest European prevalence was reported for Norway with 1.1% (2 years). In China, sensitization rates varied from 0.2 to 0.8% [59•].

Prevalence data using diagnostic food challenges are scarce. Open food challenges revealed fish allergy in Europe at 0.1% for adults (22 years old, Denmark) and children (6 years, Finland) vs 0.2% for Southeast Asia children (3–7 years, Thailand) [60, 61, 65]. Double-blind, placebo-controlled food challenges confirmed rates of 0.2% for Denmark and 0.3% for Iceland while other studies found no fish allergy in their target cohorts (e.g. UK) [68–70] . To conclude, an updated view on epidemiology of food allergy to fish points to a broad range of prevalence, up to 7.0% by self-report, up to 2.9% by determination of allergic sensitization (skin, IgE reactivity) and up to 0.3% by oral provocation tests [59•].

Fish allergy is also known to be relevant in the occupational context leading to important clinical symptoms such as skin rash, allergic rhinitis or asthma which might even force individuals to change their professional field of activity [71, 72]. The exposure to high levels of fish allergen in the working environment constitutes a continuous source and risk for allergic sensitizations [73]. In single studies, allergic asthma has been reported to occur in up to 8% and allergic contact eczema in up to 11% of employees in fish-processing working areas [55].

Beyond the working environment, fish allergens may also be present in the domestic setting. Relevant quantities have been reported in a study quantifying fish allergens in mattress dust of Norwegian houses [74]. For peanut allergy, peanut residues in households are known to correlate with consumption and for now, are considered as prime candidates to trigger allergic sensitization [75]. To which extent, this non-occupational, home exposure to fish allergens, especially in children with impaired skin barrier function, might play a role in allergic sensitization during early childhood is not known.

Fish-allergic patients usually start to react to fishes being part of the local diet [76, 77]. Most subjects experience clinical cross-reactions to a multitude of fish species. This consensus opinion on cross-reactivity is omnipresent in the literature, although mostly based on clinical reports and not on diagnostic in vivo testing.

Using questionnaires, a European study reported that 71% (44/62) of the patients experienced high cross-reactivity while 29% had low cross-reactions to different fishes [3•]. A Japanese study showed that 88% (84/95) of the patients reacted with many species and that study participants were not satisfied with the food labelling that did not allow to always identify the ingested species [78].

Using IgE based analysis, a multitude of studies have been performed in the context of fish allergy. Extensive in vitro cross-reactions, nearly 100%, were demonstrated during these investigations, both for IgE reactivity to extracts as well as for IgE reactivity to the allergen components [78–82].

Only a few studies have been published using diagnostic food challenges, reporting on limited clinical cross-reactivity such as 33% (3/9) patients or 27% (3/11) patients reacting with all investigated fishes [83, 84]. Recently the first double-blind, placebo-controlled food challenge (DBPCFC) trial with different fish species was published shedding light into clinical cross-reactions of fish-allergic patients [85•]. This trial showed that more than 50% of all 35 participants had no objective symptoms and around 30% had subjective tolerance to at least one of the three investigated fish species.

Since a few years, beyond extensive cross-reactivity to many fishes, it is now well established that patients may tolerate specific fishes [86]. Although prevalence data for patients with specific clinical reactivity are not exactly established, the phenotypic characteristics have been described based on smaller case series or single case reports. Those patients might react either to fish of the same or closely related taxonomic families such as salmonids, or they might react to single species such as to cod or sole [87–91]. Future studies such as the recently published DBPCFC-trial will contribute an advanced understanding of clinical—both broad or limited—cross-reactions in fish-allergic patients [85•].

Cross-reactions between fish and other foods are known since a decade. So far, no cross-reactivity is known for fish and shellfish or molluscs, although the allergenicity of fish tropomyosin, as a putative cross-reactive allergen to the major shrimp allergen, has been discussed [92]. Clinical cross-reactions between fish and frog meat might occur, the prevalence of fish-allergic patients with adverse symptoms upon ingestion of frog legs is unknown [93, 94]. More recently, a new clinical cross-reactivity has been described, the ‘fish-chicken syndrome’ [95, 96]. Patients with either fish allergy or genuine chicken meat allergy might become cross-reactive to the other food as IgE antibodies recognize homologue allergens being present in both chicken and fish muscle tissue.

## Fish Allergens

### Major Allergen Families

Fish allergens belong to four distinct protein families, parvalbumins, enolases, aldolases and collagen (Table 1).

Parvalbumins are the most potent fish allergens, leading to primary sensitization followed by elicitation of adverse reactions upon ingestion or inhalation [97]. They are low molecular weight proteins (10–12 kDa) that are highly stable (e.g. upon heating, enzymatic digestion) and bind bivalent ions such as calcium or magnesium [98]. Parvalbumins belong to the ‘EF hand’-protein family as the ion-binding is affected by structural motifs, which are characteristic for this family [99]. The ion-binding has been shown to be essential for the stability of the molecule and thus, in a wider sense to be related to the allergenicity of the molecule [100, 101]. The physiological role of parvalbumins in the muscle cell has been related to a calcium-buffer function during contraction and relaxation [102, 103]. Two distinct lineages, α- and β-parvalbumins are known while the β-isoform is commonly found in the muscle of bony fishes.

Enolases are ca. 50 kDa enzymes involved in the glycolysis of the muscle cells with two magnesium-ions bound in their catalytic binding site [104•]. A specific protein fold, barrel-like, classifies enolases as members of the ‘TIM barrel’-protein family. Among three known isoforms (α, β and γ), β-enolases are expressed in vertebrate muscles. Fructose 1,6-bisphosphate aldolases are ca. 40 kDa enzymes with a functional role in the cellular energy metabolism. Similarly to enolases, aldolases have also a TIM barrel-characteristic protein fold. Vertebrates express three aldolase isoforms (A, B and C), aldolase A is found in the muscle tissue. Both enzymes, enolases and aldolases, have a lower stability towards effects of food processing than parvalbumins [3•, 95].

Being essential for the structural support, collagen is commonly expressed in the skin, bones and connective tissues. It is a ca. 300 kDa-molecule of rod-shape which is composed of a triple-helical structure [104•]. Each protein chain has about 1000 residues with high repetitions of the amino acids glycine, proline and hydroxyproline. The hydrolysis product (acid, alkaline) of collagen is fish gelatin which is commonly used as a substitute for gelatins from mammalian origin [105].

### Cross-Reactive vs Specific Fish Allergy Markers

#### Parvalbumins

Most fish-allergic patients have IgE antibodies directed against parvalbumins [106]. Depending on the study design (selected parvalbumin, study group), the general prevalence seems to range between 70 and 100% [3•, 85•]. Parvalbumins exhibit common IgE epitopes which have been proposed to be located in the highly conserved, calcium-binding regions of the molecule [107]. In vitro, cross-reactivity has been shown for parvalbumins from commonly consumed fishes such as cod, salmon and tuna [2••]. In the diagnostic context, this high cross-reactivity has been concluded to bear a high sensitivity but a low test specificity [85•]. A recent study showed that nearly all participants had IgE reactivity against all tested parvalbumins while in ca. 30% of the individuals, oral tolerance (DBPCFC) was found to at least one of the tested fishes.

Parvalbumins of the α-lineage are found in muscles of vertebrates and amphibians while β-parvalbumins are present in the muscles of bony fishes. As of their high homology to the human homologue, parvalbumins from the α-parvalbumins were concluded to be non-allergenic [99]. This dogma was revised more recently based on the finding that chicken parvalbumin, an α-isoform, was identified as an important chicken meat allergen with cross-reactivity to the fish homologue [95]. The overall degree of cross-reactivity between fish β- and chicken α-parvalbumins is estimated to be low to moderate but further studies are needed to clarify the level of cross-reactivity. Cartilaginous fishes, such as rays and sharks, are another source of muscle α-parvalbumins. The clinical cross-reactivity between bony and cartilaginous fishes is not yet well analysed. However, dogfish, a small shark species, has been suggested as a low allergenic fish as it displays a limited IgE cross-reactivity between involved α- and β-parvalbumins [108].

For many years, IgE cross-reactivity to parvalbumins of the β-lineage was postulated to play a key role. Now, species-specific epitopes have been identified on single parvalbumins [88]. Patients with clinical monosensitivity to salmonid fishes seem to have an IgE repertoire only recognizing salmon parvalbumin. Involved IgE epitopes have been proposed in protein regions distant from the calcium-binding site, for salmon but also for other parvalbumins involved in monospecific fish allergy [76, 91]. Whether species-specific parvalbumin epitopes or rather—independent of the molecule—fish-specific parvalbumin contents cause variable allergenicity of different species, still needs to be resolved in the future [109, 110].

#### Enolases and Aldolases

When first discovered as fish allergens, the prevalence of IgE reactivity to enolases was found to be ca. 63% and to respective aldolases ca. 50% [3•]. High cross-reactivity between cod, salmon and tuna homologues has been shown. A follow-up study reported variable prevalence rates for the allergens from different fishes, 70 and 65% for the cod enolase and aldolase, 15 and 30% for the salmon enolase and aldolase as well as 20 and 10% for the mackerel enolase and aldolase [85•]. This might be explained by a lower inter-species IgE cross-reactivity of salmon and mackerel allergens compared to the cod homologues or by a high primary sensitization of the study participants to cod. However, IgE cross-reactivity between fish enolases and aldolases seems to be clearly lower than cross-reactivity among parvalbumins. Therefore, it has been proposed that sensitization to those allergens most likely reflects true fish allergy. This limited cross-reactivity has been also shown in a case series on clinical monosensitivity to cod which could be successfully linked to specific IgE recognition of cod enolase/aldolase at the diagnostic in vitro level [87]. Fish enolases and aldolases might also cross-react with homologues from chicken meat [95]. However, cross-reactivity has been only addressed in a cohort of patients with fish and chicken meat allergy so that the general degree of cross-reactions is not well understood so far.

#### Collagen

The prevalence of IgE reactivity might strongly vary according to regional eating habits. A European study reported ca. 20% of IgE recognition in fish-allergic patients where the relevance for clinical reactivity is still not resolved [3•]. IgE-binding rates seem to be higher in Asia where raw fish consumption is common. More than 30% of Japanese patients have specific IgE to fish collagen [78, 111]. Recently, a high level of cross-reactivity (87–98%) has been reported between collagen from mackerel and collagens from 22 other fish species [111].

## Conclusion

Animal dander and food extracts are still widely used in allergy diagnosis. As long as the list of specific allergen components is not complete, extracts are valuable screening tools. However clinicians have to be aware that extracts do not allow to determine the primary sensitization source due to the presence of different cross-reactive allergens. Some of the available allergen components are regarded as marker allergens for a specific sensitization. The ideal marker allergen should be recognized by 100% of the sensitized patients and not display any cross-reactivity to other allergens.

Several components are regarded as marker allergens for cat or dog allergy. Fel d 1 is the dominant cat allergen and a marker of primary sensitization to cat. Can f 1 and Can f 5 are the dominant marker allergens for dog, although Can f 1 is cross-reactive with Fel d 7. Can f 2 is a minor allergen and should be tested in combination with Can f 1 and Can f 5. Cross-reactive allergens may be useful as markers of cross-reactivity. Patients with moderate to high IgE reactivity to serum albumins should be advised that they may experience clinical symptoms to different furry animals. They are also potentially at risk of developing symptoms upon ingestion of unboiled milk and raw or medium-cooked meat.

Parvalbumin, enolases, aldolases and collagens are important fish allergens. IgE-reactivity patterns might vary according to regional eating habits and local diets. Parvalbumin seems to be a marker allergen for clinical cross-reactions and a diagnostic test characterized by high sensitivity but low specificity. The presence of IgE to enolases and aldolases, in addition to specific clinical symptoms, might reflect true fish allergy. The relevance of fish collagen as a diagnostic component is not yet resolved but it is expected to be of relevance for patients form specific regions such as Japan. Overall, a complete panel of fish allergens might be required to advance component-based diagnosis of fish allergy in order to lower the need for oral provocations.

The use of components allows to distinguish a primary sensitization from a cross-sensitization. This is particularly important when immunotherapy is envisaged in order to choose the primary sensitizing allergen source for therapy. Co-sensitization to two or more allergen sources can also occur and this should be determined by the use of marker allergens. Unfortunately good markers of mammalian allergens are only available for cat and dog. Commercially available components for horse, mouse and rat are cross-reactive. No components are available at all for small pet animals. The number of allergens available for fish is very limited and should be extended in order to propose a panel of components from main fish families.
